# The onset of intermetatarsal bursitis in patient with rheumatoid arthritis — Case report of surgical treatment

**DOI:** 10.1016/j.ijscr.2024.109537

**Published:** 2024-03-16

**Authors:** Yuya Kimura, Ichiro Kikkawa, Hideharu Sugimoto, Shigeo Kawai, Katsushi Takeshita

**Affiliations:** a Department of Orthopedic Surgery, Nasu Chuoh Hospital, Otawara, Japan; b Department of Radiology, Shin-Kaminokawa Hospital, Kaminokawa, Japan; c Department of Pathology, Tochigi Medical Center Shimotsuga, Tochigi, Japan; d Department of Orthopedic Surgery, Jichi Medical University, Shimotsuke, Japan

**Keywords:** Rheumatoid arthritis, Intermetatarsal bursitis, Case report

## Abstract

**Introduction:**

Mono-arthritis and intermetatarsal bursitis according to rheumatoid arthritis aren't aware among general orthopedic surgeon. This report describes a case of surgical treatment of intermetatarsal bursitis.

**Presentation of case:**

A 50-year-old female presented with three years of metatarsophalangeal joint pain and deformity. MRI showed bursitis and synovial proliferation around the joint. Synovectomy reduced pain and foot deformity. After surgery, the patient was administered methotrexate.

**Discussion:**

There were previous studies reporting intermetatarsal bursitis associated with rheumatoid arthritis, few case reports were found in which surgery and pathological examination were performed.

**Conclusion:**

Intermetatarsal bursitis is common for patients with rheumatoid arthritis. Early diagnosis and early appropriate treatment is necessary.

## Highlights

• Intermetatarsal bursitis is common in patients with rheumatoid arthritis.
• Early appropriate laboratory test and magnetic resonance imaging help us diagnose.
• It is important for general orthopedic surgeons to keep in mind that intermetatarsal lesion is an early symptom of rheumatoid arthritis.

## 1 Introduction

Rheumatoid arthritis (RA) affects joints and can cause significant impairments in daily life. The foot is often the first site of symptoms and foot problems are strongly related to RA [1]. Approximately 20 % of patients with RA present initially with foot and ankle symptoms, and most patients will eventually develop foot and ankle symptoms [2]. The appearance of foot lesions is very common in RA, but cases of mono-arthritis and intermetatarsal bursitis (IMB) aren't aware among general orthopedic surgeon. We experienced surgical case of IMB lesion with RA. This study has been reported in line with the SCARE criteria [3].

## 2 Case report

**Fig. 1:**
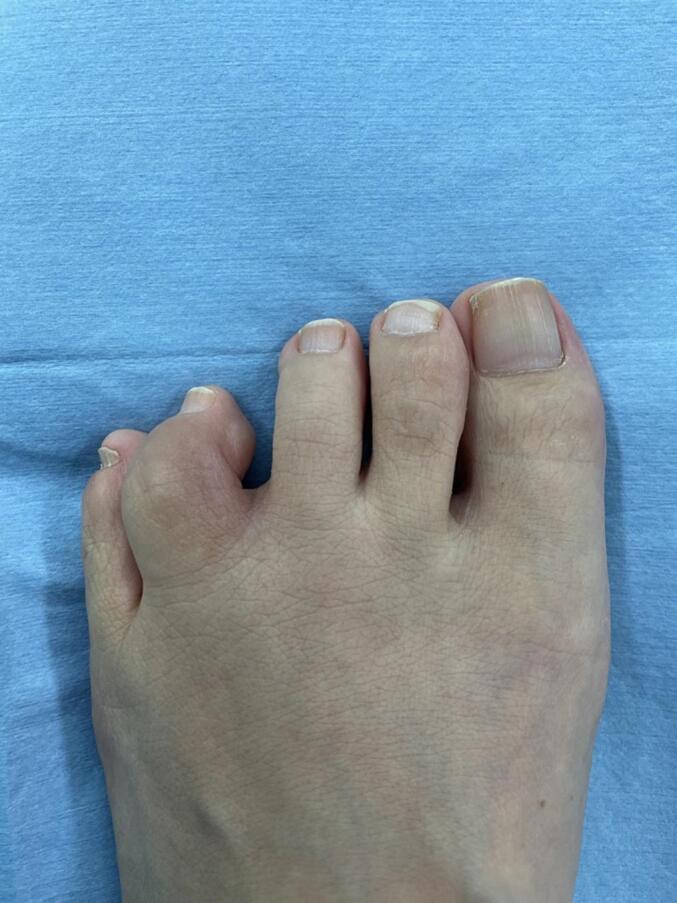
Preoperative figures: swelling and subcutaneous hemorrhage around 4th MTP joint of left foot.

**Fig. 2:**
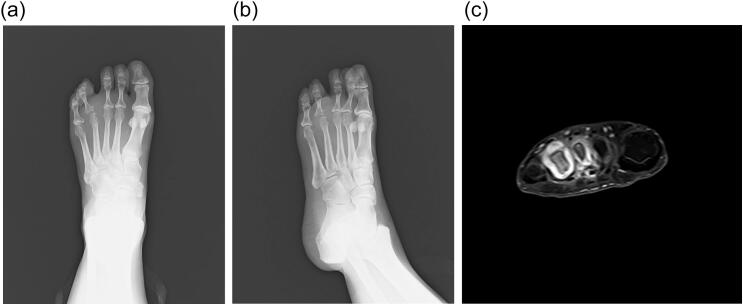
Foot involvement in a 50-year-old female. Anteroposterior (a) and oblique (b) plain radiographs of the left foot. Axial T1-weighted fat suppressed contrast enhances MRI image (c) showed synovial proliferation and fluid around 3rd and 4th metatarsals.

**Fig. 3:**
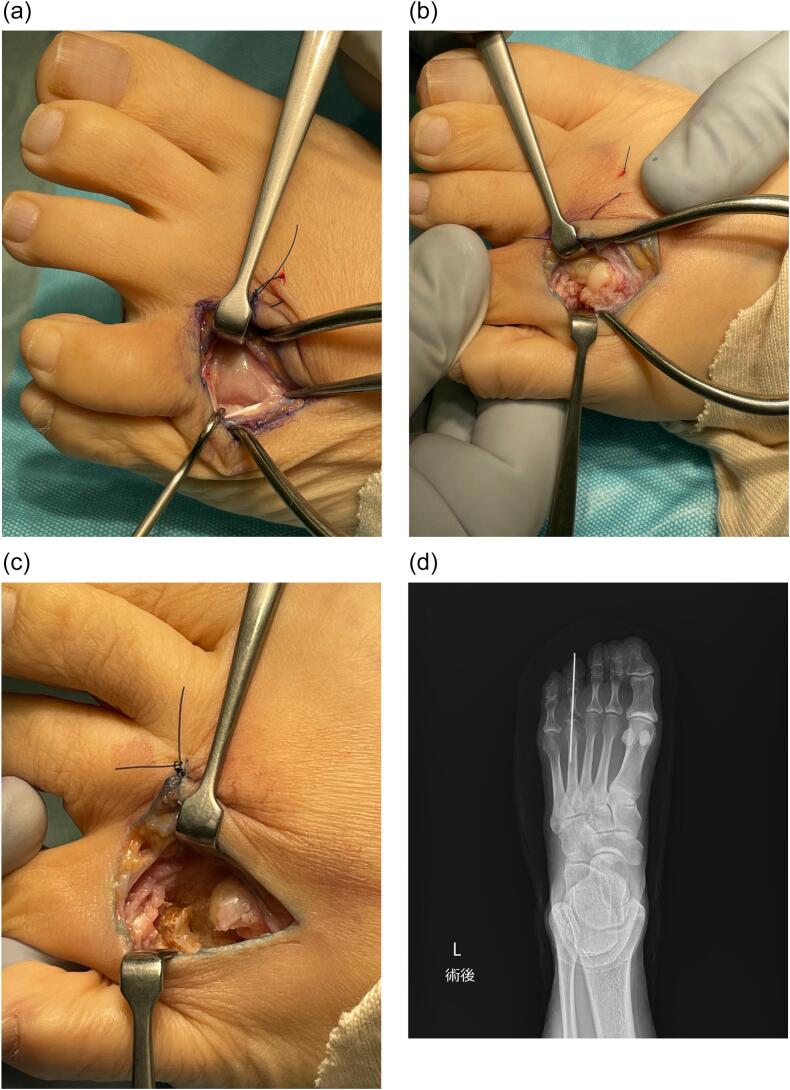
Intra-operative findings. Enlarged MTP joint capsule (a), destruction of articular cartilage and subchondral bone (b), after synovectomy (c), and postoperative X-ray (d).

**Fig. 4:**
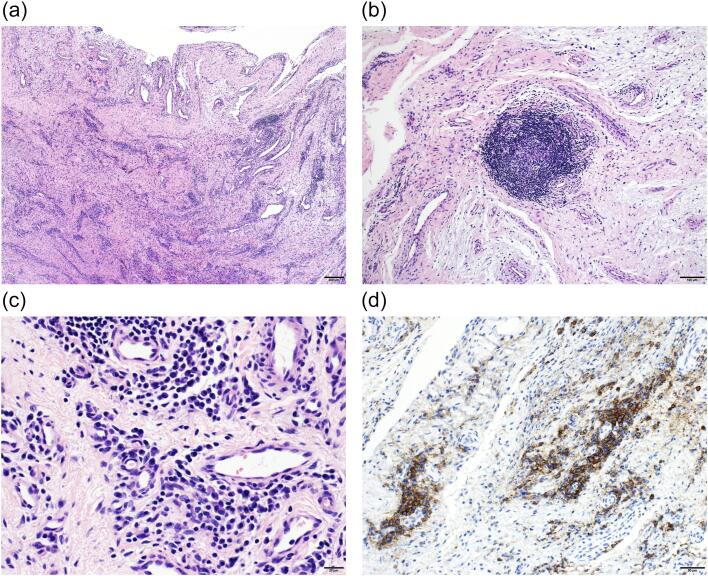
Section of the MTP joint synovium. (a and b) show hematoxylin and eosin (H&E) staining at low magnification. (c) shows H&E staining at high magnification. (d) shows CD138 immuno-staining. There was infiltration by inflammatory cells including plasma cells with blood vessel proliferation and formation of lymphoid follicle. CD138 immuno-staining which is characteristic of plasma cells was positive. Findings were consistent with chronic synovitis consistent with rheumatoid arthritis.

**Fig. 5:**
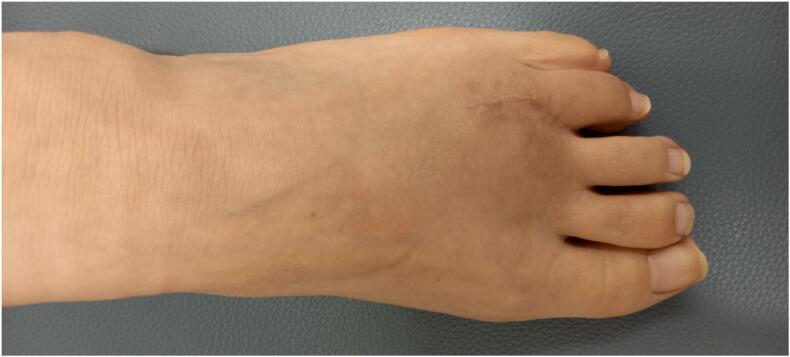
Two months postoperative figure.

A 50-year-old female presented with three years of metatarsophalangeal (MTP) joint pain. She has no family history of RA. She visited our hospital with a chief complaint of foot pain and swelling 3 years ago. Morton's disease was initially suspected. She stopped visiting our hospital because pain did not get better. 3 years after, she was referred from clinic to our hospital because of the foot deformity, subcutaneous hemorrhage, and bone erosion image on the X-ray. On physical examination, she had pain and swelling on MTP joint of forth toe (Fig. 1). The MTP joint had 0° of extension and 30° of flexion. Pain on motion was observed at that time. Laboratory tests revealed low levels of inflammatory markers and positive serological markers for RA: C-reactive protein (CRP) was negative, rheumatoid factor (RF) was 29.3 IU/mL, and anti-cyclic citrullinated peptide (ACPA) was 496.6 IU/mL. She scored 6 points according to the 2010 American College of Rheumatology/European League Against Rheumatism (ACR/EULAR) classification criteria and was classified with RA. X-ray showed bone erosion of metatarsal (Fig. 2a and b). MRI showed synovial proliferation and fluid around 3rd and 4th metatarsals (Fig. 2c). Because of difficulties in diagnosis, disease-modifying anti-rheumatic drugs were not administered prior to surgery, only non-steroidal anti-inflammatory drugs were administered, but there was no improvement in pain. She had pain due to lateral dislocation of proximal phalanx of foot and was performed synovectomy for diagnosis and treatment. Enlarged MTP joint capsule was observed (Fig. 3a). Incision through the joint capsule revealed synovial proliferation (Fig. 3b). After synovectomy, we found destruction of articular cartilage and subchondral bone (Fig. 3c). Joint instability due to periarticular tissue destruction was observed, so MTP joint was fixed by using Kirschner-wire (Fig. 3d). On pathological examination, there was infiltration by inflammatory cells including plasma cells with blood vessel proliferation (Fig. 4a and c) and formation of lymphoid follicle (Fig. 4b). CD138 immuno-staining was positive (Fig. 4d). CD138 is a member of the syndecan family of type I transmembrane proteoglycans. CD138 is highly expressed on plasma cells which is characteristic for RA [4]. Pathological findings were consistent with chronic synovitis consistent with rheumatoid arthritis. Surgical wound had healed in two weeks. K-wire was removed one month after surgery, and she could walk with a little pain. The MTP joint had 20° of extension and 40° of flexion postoperatively. Postoperative figure is shown (Fig. 5). After that, she was administered methotrexate at 4 mg per week. The pain has been getting better after administering methotrexate. 8 mg per week of methotrexate is still being administered to inhibit the progression of RA.

## 3 Discussion

RA is chronic inflammatory disease especially in small joints. Our patient was initially suspected Morton's disease, but bone destruction has appeared a few years later, and tumor was suspected at the initial visit of our hospital. The differential diagnosis in these cases may be tumor such as Morton's disease, or other arthritis. On MRI, Morton's neuromas are within the intermetatarsal space and centered in the neurovascular bundle on the plantar side of the deep transverse metatarsal ligament [5]. Morton's neuromas are low to intermediate signal intensity on MRI, reflecting the fibrosis around the nerve [6]. On the other hand, main MR findings were distension of the intermetatarsal bursa with increased signal intensity on T2-weighted and post-contrast fat saturation T1-weighted images [7]. MRI of our patient has synovial proliferation and fluid around metatarsals which is RA-like, not Morton's disease-like. IMB can potentially be the first manifestation of different rheumatological diseases [7].

The probability of the presence of IMB in RA patients is up to 90 % according to prospective ultrasound studies [8,9]. Although there were previous studies reporting IMB lesion associated with RA, few case reports were found in which surgery and pathological examination were performed. Past studies with RA showed progression to poly articular RA over 3 to 4 years in most cases [10]. In our case, RA first developed in the IMB lesion and gradually progressed to the MTP joint, leading to bone destruction in a few years. Most general orthopedic surgeons are probably unaware that IMB lesions appear as early lesions in RA, so some RA patients might be misdiagnosed as another disease. The new point of this study is that it gives general orthopedic surgeons an option for the diagnosis and treatment of rheumatoid foot lesions. It is important for general orthopedic surgeons to keep in mind that IMB lesion is an early symptom of RA. We believe that this will make it possible to diagnose RA earlier, start treatment earlier, and prevent joint destruction.

## 4 Conclusions

IMB are common in RA patients. If left untreated, it can lead to joint deformity due to cartilage and bone destruction. When foot lesion like IMB is seen, RA should be kept in mind, and laboratory examination and MRI may be helpful for early diagnosis and early appropriate treatment is necessary for patients.

## Ethical approval

Ethical approval is not required for this study design in our institution.

## Funding

None.

## Author contribution

Yuya Kimura: Orthopedic surgeon in charge, data collection, drafted the initial manuscript and revised the manuscript after feedback.

Ichiro Kikkawa: Orthopedic surgeon in charge, supervised patient care and manuscript accuracy checking.

Hideharu Sugimoto: Radiologist in charge, prepared the radiological images with their description.

Shigeo Kawai: Pathologist in charge, prepared the pathological images with their description.

Katsushi Takeshita: Study design and concept, data analysis and interpretation, manuscript accuracy checking.

## Guarantor

Yuya Kimura.

## Research registration number

Name of the registry: Japanese Orthopaedic Association National Registry (JOANR).

Unique identifying number or registration ID: 091010130_311.

Hyperlink to your specific registration: https://www.joanr.org/.

## Patient consent

Written informed consent was obtained from the patient.

## Conflict of interest statement

The authors have no financial and personal relationships with other people or organisations according to this study.
